# Book Notices

**Published:** 1860-03

**Authors:** 


### Editorial.

### BOOK AND PAMPHLET NOTICES.

A Practical Treatise on Fractures and Dislocations. By Frank Hastings Hamilton, M. D., Professor of Surgery in the University of Buffalo, etc., etc. Illustrated by two hundred and eighty-nine wood cuts. Philadelphia: Blanchard & Lea. 1860. Pp. 757.

The appearance of an original work on Fractures and Dislocations, marks a new era in the history of surgery in the United States; for as Dr. Hamilton remarks in his preface, the English language does not contain, at this moment, a complete treatise on these subjects. Melguigne's work on fractures, has been translated into English, and published in this country, since the commencement of Dr. Hamilton's book, but we are not aware that the second part, on dislocations, has yet been published in French. Such a work was, therefore, a *desideratum*, and this, we feel assured, will not only supply the want, but be a starting point for a more careful study of these accidents.

It is a work of great labor; embracing not only the views of leading European authors, but those of a large number of American practitioners, collected from detached and scattered sources, such as publications in Medical journals, transactions of Societies, etc. He has also brought to bear upon the subject, numerous valuable specimens in the hands of various surgeons in this country, not heretofore employed in a way to be so generally accessible, as those in public collections.

In his labors, the author has not only manifested an industry worthy of praise, but an impartiality much more rare; and has shown that materials exist in this country for aiding in the solution of many difficult problems, if writers would but take the necessary pains to collect them, instead of adopting the easier and more usual method of resting satisfied with such as are furnished them by foreign writers.

The work is concise, judicious, and accurate; and adapted to the wants of the student, practitioner, and investigator, honorable to the author, and to the profession.

We would not, however, be understood as agreeing with Dr. Hamilton in all his views of practice. This could not be expected. Each surgeon has his own method of effecting any given object, which by skillful application he makes more useful than another method equally good, would be in his hands, and attributes, naturally, to the apparatus, results due to his skill. Numereus figures and descriptions of dressings, are found in this work which, although they have been proposed and recommended by eminent surgeons, are hardly used at the present day, even by their authors.

Thus, we are informed that Seutrin has so mollified his "immovable" dressings, as to make them differ but little from that with padded splints; and we doubt if Velpeau is tenacious of the "dextrine" bandage so popular with him in 1840. But our object is not to criticize, or to give an analysis of the work, which each one of our readers, in surgical practice, should possess. There are, however, a few points of special interest to us, which it may be of use to notice in detail. Among these, the perforation of bone in the treatment of ununited fracture is the most prominent. Dr. Hamilton's notice of the plan proposed by us, is perfectly fair, and contrasts most favorably with that of Prof. Gros, who not only says the perforation did not succeed with him, (he using a common trochar with which it is impossible to perform it,) but misquotes what we have published on the subject. Dr. Hamilton does not give us his own experience the treatment of unuited fracture; but we remember to have noticed some years since, a successful case, reported by him, treated by a method similar to our own. As several cases have come to our knowledge, of surgeons using our method, as they suppose, without success; when, in fact, they resorted to one quite different, and which in the beginning we found equally to fail; and as some like Dr. Gross have supposed that we claimed always to effect a cure, and more particularly in reply to numerous letters and applications on the subject, we repeat here what we have already published, of the results of our observations and operations, together with some additional facts necessary for their full elucidation.

We have treated, for the purpose of obtaining union, at the present date, twenty-one cases of ununited fracture.

Of these, two were treated by fixing the ends of the ends of the bones together by pieces of wire. Both were of the femur, and succeeded, but with symptoms quite severe.

Since adopting the practice of subcutaneous perforation, we have treated nineteen cases, as follows:

| | |
|---|---|
| Humurs, . . . . . . . . .4. | Ulna,. . . . . . . . . . . .3. |
| Femur,. . . . . . . . . . .5. | Tibia, . . . . . . . . . . . .5. |
| Radius, . . . . . . . . . .1. | Lower Jaw,. . . . . . .1. |

All these, except two, have been already published. One left while under treatment, and in a fair way of recovery, to place himself under the care of another surgeon, who employed the seton without success. Once it failed, as did also the the seton and resection, this was cured near three years after, and recently by the use of metallic wire through the ends of the bone. Thy rest recovered without serious accident. Two had small abscess at the point of perforation, one had slight erysipelas. This was the case alluded to as not succeeding, and the erysipelas was much more mild than that which followed the use of the resection.

This is not all our experience in the treatment of ununited fracture. Several cases of delayed union, of from twelve to sixteen weeks standing, have been treated successfully without operation. These were cases in which there was already some tendency to union. Where there is no tendency to union, after double the time ordinarily required, I resort to perforation.

In two cases of ununited fracture, I have performed amputation. One was a compound fracture of the humerus, with paralysis of the member of eighteen months standing; the other, a fracture of the tibia of eleven years standing, and much deformity, and where sloughing had taken place to such an extent from erysipelas, that during the whole of this time, cicatrization had never been completed.

In addition to these cases, we have seen and examined several ununited fractures, which we have not treated at all; some, because treatment was not desired; others, because the fracture was of ancient date, and the member considerably useful; and two, because the state of health of the subjects was not such as to admit of any operation being performed with safety.

Of the cases which were not submitted to treatment, several had already been treated by the seton without success.—One cured by boring, had also been previously treated in the same manner; and in one, we resorted to the seton after perforation had failed, as above stated. It did not succeed in any of these cases. We have not had occasion to see the treatment by seton result favorably in a single instance. We do not from this fact, reject the seton absolutely. In most of the false joints we have been able to acquire a knowledge of, on which the seton was used, it has been resorted to with so little discrimination of cases, and so little knowledge of its action, used too small or too large, or for too short a time, that they could not by any means be regarded as fair tests of its value when properly resorted to. In but one, was it used according to the directions of its author, who retained it over four months before union was completed, and twelve weeks before it commenced to take place. In the case to which we now allude, the seton was kept in the arm eleven months without success.

Dr. Hamilton's enumeration of the causes of want of union is very brief and does not put the surgeon sufficiently upon his guard against some of them—such as transportation of the patient from place to place; the application of cold or evaporating lotions, etc. These, according to our observation, are constantly followed by some delay of union.

Of the method of remedying deformities of bones, after fracture, by subcutaneous division, some examples of which, are familiar to our readers, Dr. H. does not speak; no doubt from not having noticed reports of them.

On the subject of fractures of the neck of the femur within the capsule, our author adopts the views, very nearly, of Sir Astley Cooper, and reads a lecture to the professors of the Royal College of Surgeons, London, for their want of justice towards Sir Astley. Having had occasion to examine the collection of Cooper, now in the museum of the college, and to hear and see both sides, it seems to us that the Doctor might very well have omitted the little moral lesson on that point.

On the subject of reduction of dislocations of the hip, the remarks of Dr. H. are very valuable. He quotes five instances in which the femur was fractured during efforts at reduction by extension, and two when the same accident happened from manipulation.

While his conclusions are not unfavorable to manipulation, he expresses himself with reserve concerning it. We do not by any means share his doubts of the permanent value of this method of treatment; nor do we expect that it is destined to supercede the use of extension with the pulleys. By further experience, and more careful studies on the *cadaver*, the two methods may be so combined as to render mutual service and greatly mitigate the severity of these formidable accidents.

With a recommendation to such as desire a knowledge of the subject, to "buy the book," we close this notice, proposing to return to some points of its practice and pathology, as occasion may present.

Contributions to Operative Surgery and Surgical Pathology. By J. M. Carnochau, Professor of Surgery in the New York Medical College, etc. No. Three. Philadelphia: Lindsay & Blakeston.

This is the continuation of a work unequalled among the original medical publications of this country, for the elegance and luxury of the "getting up." It is of quarto form, on fine paper, with large type, and illustrated by tinted lithographs of excellent quality.

Professor Carnochau is fortunate in being able to put his "contributions" to surgery in a form so attractive and so permanent. The present numbers contains three papers on congenital dislocations of the femur, and one on restoration of the entire upper lip.

The work is published in numbers, which appear quarterly, at seventy-five cents each. The entire work to be completed in ten numbers.

---

THERAPEUTICS AND MATERIA MEDICA.—A systematic Treatise on the Action and Uses of Medicinal Agents, including their Description and History. By ALFRED STILLE, M. D., late Professor of the Theory and Practice of Medicine in the medical department of Pennsylvania College; formerly physician to St. Joseph Hospital; President of the Pathological Society, and Fellow of the College of Physicians, of Philadelphia; Member of the Societe Medicale D'Observation, of Paris; Honorary Member of the Medical Society of the State of Rhode Island, etc. Philadelphia: Blanchard & Lea. Two Volumes. Pp. each, 900. 1860.

It is with no common satisfaction that we welcome this contribution of Prof. Stille, to the rapidly augmenting sum of knowledge, relating to remedial agents offered to the acceptance of the diligent student.

An accurate and available understanding of Therapeutics, is the very corner-stone, upon which every physician must rest the superstructure of successful practice; and yet, it is a subject usually deemed so little capable of embellishment, that study is too often neglected; many physicians passing through a long professional life, constantly wielding weapons of the action and power of which they are only partially informed. We think this work will do much to obviate the reluctance to a thorough investigation of this branch of scientific study; for in the wide range of medical literature treasured in the English tongue, we shall hardly find a work written in a style more clear and simple, conveying forcibly the facts taught, and yet free from turgidity and redundancy. There is a fascination in its pages, that will insure to it a wide popularity, and attentive perusal, and a degree of usefulness not often attained through the influence of a single work.

The author has much enhanced the practical utility of his book, by passing briefly over the physical, botanical, and commercial history of medicines, and directing attention chiefly to their physiological action, and their application for the amelioration or cure of disease. He ignores hypothesis and theory, which are so alluring to many medical writers, and so liable to lead them astray, and confines himself to such facts as have been tried in the crucible of experience.

Some idea of the style of the author, and the scope and aim of the work, may be gathered by the following brief extract from its preface:

"The strictly scientific portion of the subject embraces the consideration of medicines in their physical, chemical, and physiological relations. Of these, the first and second are described so fully and accurately in works that rank among medical classics, that it seemed unnecessary to discuss them at length in a treatise whose point of view is rather at the bedside of the sick, than in the laboratory or the lecture room. On the other hand, the action of medicines upon the sound organism of man and of the lower animals, forms an indispensable key to their curative operation in disease. The more thoroughly it is known, the more intelligible must the mode become in which medicines bring about the restoration of soundness of structure and function, and the more will the isolated facts of therapeutics tend to arrange themselves in a systematic form.

"If this division of the subject is more copiously illustrated than is usual in treatises on the Materia Medica, it may perhaps the better serve to aid the sagacious reader in explaining the operation of remedies, and to suggest new occasions for their employment. Our knowledge of the usefulness of medicines rests altogether on experience; but not upon that of any one man, however skillful, or of any age, however enlightend; their efficacy is attested by a multitude of witnesses, and is to be confirmed by time which reduces the opinions of individuals to their just value, outlives the fashions of the day, and is unmoved by the prejudices of the schools. To experience then, we must turn as the ultimate and decisive arbiter of all questions respecting the curative virtues of medicines, feeling assured that whenever the particular application of a remedy can be sustained by the testimony of the great physicians of successive ages, our employment of it possesses the highest possible sanction."

The work is in two volumes. In its mechanical execution it is every way worthy of the house by which it is issued.

Received through S. C. Griggs & Co., 39 and 41 Lake St., Chicago, Illinois.

---

Elemements of Medical Jurisprudence. By Theodoric Romeyn Beck, M. D., LL. D., Professor of Materia Medica in the Albany Medical College; Member of the American Philosophical Society; Honorary Member of the Medical Societies of Rhode Island and Connecticut; etc., etc., and John B. Beck, M. D., Professor of Materia Medica and Medical Jurisprudence in the College of Physicans and Surgeons of the City of New York; Corresponding Member of the Royal Academy of Medicine, of Paris; Corresponding Member of the Medical Society of London; etc., etc. Eleventh Edition. With Notes by an association of the friends of Drs. Beck. The whole revised by C. R. Gilman, M. D., Professor of Medical Jurisprudence in the College of Physicians and Surgeons, of New York. Philadelphia: J. B. Lippincott & Co. From S. C. Griggs & Co., 39 and 41 Lake Street, Chicago. 1860.

In a new edition of this work we recognize the appearance of an old and valued friend; in a new and improved dress, enlarged by the results of the latest experience and mental effort of its author; and also by the valuable contributions, furnished by a number of friends of Dr. T. Romeyn Beck, of recognized ability, who came generously forward to perfect this new edition of his "Medical Jurisprudence," that he had well nigh completed before his death, and to place it within the reach of the medical and legal professions. If anything could add to the favor with which it is likely to be received by the public, the names of the distinguished gentlemen who have lent their supervision and aid, to amend errors if any were found, or supply deficiencies should any be discovered, would be for it a sufficient guaranty. They are as follows:—D. Tilden Brown, M. D., resident physician, Bloomingdale Lunatic Asylum; R. H. Coolidge, M. D., Assistant Surgeon U. S. A.; Austin Flint, M. D., Professor of Clinical Medicine in the New Orleans School of Medicine; B. W. McCready, M. D., Physician Bellevue Hospital; Samuel St. John, M. D., Professor of Chemistry, College of Physicians and Surgeons, New York; John Watson, M. D., President of the New York Academy of Medicine; J. P. White, M. D., Professor of Obstetrics in the University of Buffalo; John C. Dalton, Jr., M. D., Professor of Physiology in the College of Physicians and Surgeons, New York; and upon legal questions, George Shea, Esq., Counselor at Law, and the Hon. Murray Hoffman of the New York Superior Court.

---

Transactions of the American Medical Associations.—Volume 12, 1859.

This volume is less ponderous than those which have preceded it, without being on that account more condensed or valuable.

It is becoming apparent that the workers in the profession, do not desire to present the results of their labors to the public through these volumes of "Transactions." It is to be hoped that the division of the Association into "sections"

effected at the last meeting, may have a favorable effect on its action for the future.

It may be seen by the "book notices" of our present issue, that the past and present years have already issued a number of original medical works in this country, quite unusual, and of a character to add to the reputation of the profession.—This activity is not indicated by the volume before us nor can it be said that the papers contained in it are of such a character as greatly advance the standing of the physicians of this country.

---

New Medical Journal.—The *Virginia Medical Journal* makes its appearance with the new year, under the new title of the *Maryland and Virginia Medical Journal.* J. B. McCaw, M. D., and W. C. Van Bibber, M. D., editors, with a long list of able co-editors.

We cannot but applaud this evidence of a tendency to consolidation and co-operation on the part of the active and intelligent members of the profession in these two great states which, contrast with the tendency noticeable elsewhere, to isolation and subdivision.

The editors are efficient and capable, and we shall look for increased value, in a journal heretofore under its former name and direction, one of the prominent medical periodicals of the country.

In this number we have as the leading article a description of a "New Instrument for the Treatment of Fractures of the lower extremity," by Prof. N. R. Smith, a veteran in the service.

This splint is called by its inventor the "anterior splint" and is placed, as its name indicates, on the upper instead of the under side of the member, as other angular splints are. Not having used this splint, nor even seen it applied, we are not prepared to give an opinion concerning its value, but think from the description that in certain cases of compound fracture, or where excoriations have occurred, it will be found a reliable resource.

In the notice of the Volume of Transactions of the American Medical Association, we find the following judicious remarks which we commend to our readers on account of their truthfulness, good sense, and their appropriateness at the present time:

"Always an earnest friend of this Association, and recognizing its conservative influence upon our profession, we have never been one of those who preferred to point out its failures, rather than to dilate upon the usefulness of its results. The warmest advocate of the Association, however, must confess, that it fails in each annual reunion to equal, much less excel, its efforts of the preceding year. Its committees have, one after another, been found wanting, until, as in the present instance, even the standing committees failed in their duty, and we have no report on the present condition of medical literature or education in the United States.

"Another ominous prognostic may be noticed in the refusal of the prize to any of the applicants; for surely there must be apathy among *forty thousand* physicians, if an essay coming up to the modern standard heretofore assumed by the Association could not have been prepared for the occasion.

"Need we add, that each returning year shews a smaller attendance of the profession upon this their national congress, and each year—most vital of all—the volume of the Transactions in interest and value.

"These facts make us disposed to think seriously upon the course to be pursued hereafter by all who wish to perpetuate this important organization, and with the hope of throwing out suggestions which may prove useful at the coming meeting, we take this opportunity to express our opinions on this question. We may reasonably expect to meet many of the leading men of the country at New Haven in June, and we anticipate that some modifications of the existing arrangements will be discussed.

"We regard the two vital defects in the present system of management to be as follows: 1st. The Association has never been a scientific working body, but a debating society, confining itself to the discussions of constitutional and ethical questions, attempting to pull down or build up colleges, or to execute impracticable schemes of reform, and generally concluding with a grand jollification which promoted mirth, but gave not a page to the forthcoming volume."

We have in conclusion a small reclamation to make of the "Treatment of Indolent Ulcers by the Vapor of Iodine," which is in the number before us, credited to the *N. A. Med. Chic. Review*. This treatment originated in the U. S. Marine Hospital, at Chicago, under our care, and was first published in a recent number of the *Chicago Medical Journal*. Notwithstanding the high authority for taking from those who have little and giving to those who have much, we object to the application of the practice in this and similar cases.

---

Louisville Medical Journal, Edited by Thomas W. Colescott, M. D., Louisville, Ky: Vol. 1, No. 1.

This medical journal, edited by one already well known to the fraternity by his connection with the *Louisville Medical Journal*, made its appearance at the commencement of the new year. The number before us is well got up, both editorially and typographically, and from the names of the contributors to the present there can be no doubt of the value and interest of the future numbers. Pecuniarily we should suppose that a new journal in Louisville would not be a promising enterprise. There are already eleven medical periodicals issued in the Northwest, including those of Cincinnati, Louisville, and St. Louis, where three or four, or even one might answer the purpose of the profession, but this number only indicates the zeal and enterprise of physicians in this region, and at present that concentration which seems desirable is apparently impracticable.

The first article in the *Louisville Medical Journal* is by Prof. Goldsmith—a report of a case of traumatic aneurism of the femoral artery, in which the common iliac artery was tied by penetrating the peritonial cavity. Death occurred on the fifth day after the operation. The cause of the fatal result, in the opinion of Prof. Goldsmith, was the "constitutional contamination produced in consequence of the absorption of decomposed blood clots."

He expresses the opinion also that the mortality which has attended the operation for the cure of large false aneurism by the so-called Hunterian operation, is much greater than that after treatment by the old method of ligature above and below the tumor, with evulsion of the blood clots.

---

Introductory Lectures and Addresses on Medical Subjects. Delivered chiefly before the Medical Classes of the University of Pennsylvania. By George B. Wood, M. D., LL. D., etc. Philadelphia: J. B. Lipincott & Co. 1850. Pp. 460.

The author in his preface gives the following reasons for the publication of these addresses:

"Being about to withdraw from scholastic medical teaching, the author conceives that this may be a proper occasion for publishing, in a connected form, the introductory lectures and addresses, relating to medicine, which he has at various times delivered. Most of them have been already printed separately by the several classes or societies before whom they were respectively read; but some of them now appear in print for the first time. Representing, as they do, the views and sentiments of one long devoted to the medical profession, and compelled, by the necessities of his position, to observe, investigate, and reflect upon the concerns of that profession in all its different relations, scientific, practical, ethical, and historical, they can scarcely fail to contain lessons, which may be made useful to the student and young practitioner. This consideration may, perhaps, be received as a sufficient excuse for their publication; but the author confesses that he has also other views. He wishes to bring himself again to the memory of the many physicians, some of them now no longer young, who have listened to his instructions during their years of pupilage, and to leave with them a memento, by which, when he shall be no more personally among them, they may now and then recall him to mind, with kindly recollections of former intercourse."

These sentiments we are sure will be cordially reciprocated by the physicians of this country, whose respect, admiration, and kind feeling will follow Dr. Wood into his retirement, and to whom this volume is a timely and acceptable offering.

